# Assessment of demographic characteristics and histopathological pattern of thyroidectomies patients in a resource-limited setting: a retrospective cross-sectional study

**DOI:** 10.11604/pamj.2022.43.213.36495

**Published:** 2022-12-30

**Authors:** Saif Ghabisha, Faisal Ahmed, Qasem Alyhari, Saleh Al-wageeh, Menawar Dajenah, Abdulfattah Altam, Waleed Aljbri, Abdullah Al-Naggar, Ebrahim Al-shami, Zakaria Al-mekhlafy, Mohamed Badheeb

**Affiliations:** 1 Department of General Surgery, School of Medicine, Ibb University of Medical Sciences, Ibb, Yemen,; 2 Department of Urology, Urology Research Center, Al-Thora General Hospital School of Medicine, Ibb University of Medical Sciences, Ibb, Yemen,; 3 Department of General Surgery, School of Medicine, 21 September University, Sana'a, Yemen,; 4 Department of Anesthesiology, Al-Thora Modern Hospital, Faculty of Medicine, Sana´a University of Medical Sciences, Sana´a, Yemen,; 5 Department of Internal Medicine, Faculty of Medicine, Hadhramaut University, Hadhramaut, Yemen

**Keywords:** Complications, goiter, histopathology, thyroid disease, thyroidectomy

## Abstract

Thyroid disorders are prevalent among Yemenis. However, there is limited data regarding thyroid disease burden, surgical intervention outcomes, and predictive factors in our country. This study aims to review the indications, histopathology, and complications of thyroid surgery in a resource-limited setting where the management is provided primarily by general surgeons. A retrospective study between Jun 2010 and March 2019 included 246 cases who underwent thyroid operations for a thyroid disorder in Al-Nasr Hospital, Ibb, Yemen. The patient's demographic characteristics, operative findings, complications, fine needle aspiration biopsy (FNAB) results, final pathology, and outcomes were gathered and analyzed. The mean age was 41.60± 8.31 years. The prevalence was high (30.1%) in the age group of 31-40 years and females (87.8%) with a female-to-male ratio of 7.2: 1. The main indication for thyroidectomy was compressional symptoms (35%), and the main preoperative cytology findings were multimodular goiter (89%). There was thyroid cancer in 18(7.3%) patients, and the most type was follicular thyroid carcinoma (FTC) in 9 patients. The most typical type of surgery was near-total thyroidectomy in 186 (75.6%) patients. Complications were presented in 47 patients (19.1%), and total mortality was observed in 5(2.03%) patients. Intraoperative bleeding was the most typical complication in 36 (14.6%) patients. The sensitivity, specificity, and accuracy of FNAB were 96.34%, 44.44%, and 96.34%, respectively. Fine needle aspiration biopsy (FNAB) was not precise enough in diagnosing FTC with a sensitivity of 55%. Our result showed a considerable rate of postoperative complications of thyroid surgery, and thyroidectomy may be a viable option even in a resource-limited setting or performed by general surgeons.

## Introduction

Thyroid disorders are among the most clinically encountered conditions. These are primarily caused by alterations in hormonal production, thyroid gland enlargements (goiter), or both [1]. The presentation of thyroid disorders varies based on the underlying etiology, with goiter, hypothyroidism, hyperthyroidism, thyroiditis, and neoplasms accounting for most of their presentation [1]. On many occasions, thyroidectomy is one of the most frequently performed procedures done globally by various surgeons, including general, endocrine, thoracic, head and neck surgeons, and otolaryngologies [2]. Studies on the incidence of thyroidectomy and its consequences are pretty sparse in our region due to inadequate resources despite its importance. In this study, we aimed to review the indications, histopathology, and post-surgical consequences of thyroidectomy at Al-Nasr Hospital, Ibb, Yemen, in the context of limited resources and general surgeons as the sole operators.

## Methods

**Study design:** this is a retrospective cross-sectional study between Jun 2010 and March 2019, including 246 cases operated upon for a thyroid disorder in Al-Nasr Hospital, Ibb, Yemen.

**Inclusion criteria:** all patients whose undergone thyroid surgeries, regardless of the type of surgery and the disease, in our center (Al-Nasr Hospital, Ibb, Yemen) during the study period were included.

**Exclusion criteria:** patients with incomplete information, concurrent surgery in the same hospitalization, or operated in other hospitals were excluded.

**Data collection:** the demographic characteristics of the patients, including age, gender, the indication of surge, primary diagnosis, method of surgery, duration of hospitalization, FNAB result, histopathologic diagnosis, intraoperative complications, immediate post-surgical complications, and mortality, were gathered from the patient's medical profiles. Additionally, the sensitivity, specificity, and accuracy of FNAB were compared to the postoperative pathological reports.

**Statistical analysis:** it was performed using SPSS (IBM SPSS, version 22, Armonk, New York: IBM Corp). Descriptive statistics for variables were calculated as mean and standard deviation. For reporting frequencies, descriptive analysis was used. Standard statistical formulas were used to calculate sensitivity and specificity.

**Ethical approval:** the study received ethics approval from Ibb University, Ibb, Yemen, and the Medical Research Ethics Committee.

## Results

The patient's characteristics are summarized in Table 1. Two fourty six (246) cases underwent thyroid operations with a mean age of 41.60± 8.31. The prevalence was high (30.1%) in the age group of 31-40 years, followed by the age group of 41-50 years (27.6%). Females were more affected than males (87.8% vs. 12.2%), with a female-to-male ratio of 7.2: 1. The commonest preoperative cytology finding was multimodular goiter in 219 (89%) patients. There was thyroid cancer in 18(7.3%) patients, and the most type was follicular thyroid cancer (FTC) in 9 patients. The most typical symptoms (indication for thyroidectomy) were compressional symptoms in 86 (35.0%) patients. The most familiar type of surgery was near-total thyroidectomy in 186 (75.6%) patients. Complications were presented in 47 patients (19.1%), and total mortality was observed in 5 (2.03%) patients. Intraoperative bleeding was the commonest complication presented in 36 (14.6%) patients, including postoperative hematoma in 10 (4.06%) during admission with respiratory compromise or obstruction that necessitates re-intervention or evacuation. Recurrent laryngeal nerve palsy was seen in 14 (5.7%) patients, which was bilateral in 3 (1.2%) patients requiring tracheostomy. Furthermore, postoperative wound infections were presented in 12 (4.87%) patients, and transient hypocalcemia was presented in 12 (4.87%). Most of the patients, 188 (76.4%), were hospitalized for 2 to 4 days. The sensitivity, specificity, positive predictive value, and accuracy of FNAB were 96.34%, 44.44%, 100%, and 96.34%, respectively. Fine needle aspiration biopsy was not precise enough in the preoperative diagnosis of FTC (55%) (Table 2).

**Table 1:** patient's characteristics (N=246)

Variables	N (%)
Age (year)	41.60± 8.21
**Gender**	
Male	30 (12.2)
Female	216 (87.8)
**Living area**	
Urban	74 (30)
Rural	172 (70)
**Type of operation**	
Near-total thyroidectomy	186 (75.6)
Total thyroidectomy	28 (11.4)
Unilateral lobectomy + Isthmus resection	27 (11.0)
Nodulectomy	5 (3.0)
**Complications**	
Intraoperative hemorrhage	36 (14.6)
Recurrent laryngeal nerve palsy	14 (5.7)
Postoperative wound infections	12 (4.87)
Transient hypocalcemia	12 (4.87)
Need for reoperation	10 (4.06)
Needed for tracheostomy	3 (1.2)
Death	5 (2.03)
**Hospital stays**	
2-4 days	188 (76.4)
5-7 days	48 (19.5)
More than 7	10 (2.03)

**Table 2:** the accuracy between fine needle aspiration biopsy and final pathology results (N= 246)

Final histopathology	Fine needle aspiration biopsy, N (%)					
	Multi-nodular goiter	Adenoma	Follicular	Papillary	Lymphoma	Total
Multi-nodular goiter	219 (98.2)	4 (1.8)	0 (0.0)	0 (0.0)	0 (0.0)	223
Adenoma	0 (0.0)	5 (100.0)	0 (0.0)	0 (0.0)	0 (0.0)	5
Follicular	0 (0.0)	4 (44.4)	5 (55.6)	0 (0.0)	0 (0.0)	9
Papillary	0 (0.0)	0 (0.0)	0 (0.0)	7 (100.0)	0 (0.0)	7
Lymphoma	0 (0.0)	0 (0.0)	0 (0.0)	0 (0.0)	2 (100.0)	2
Total	219	13	5	7	2	246

## Discussion

This study found that multinodular goiter is the most preoperative cytology finding. Similarly, Al-wageeh *et al*. evaluated thyroid disorders histopathologically and reported that multinodular goiter was the most prevalent preoperative cytology result [3]. These results are consistent with previous findings that goiter is the most pervasive thyroid condition affecting young women in their teens and twenties [4]. Iodine-deficient regions are rampant with nodular goiter; a greater proportion of multimodular goiter in our study's representative areas may imply an iodine deficit. Establishing the causes and pathophysiology in our community may necessities further studies. In this research, the female prevalence of thyroid illnesses over men was found, making a 7.2: 1 female-to-male ratio, similar to Al-wageeh *et al*. study [3]. Thyroid diseases affect women more than men, owing to thyroid disorders' autoimmune nature and changes in thyroid function during pregnancy [4]. The main indications for thyroid surgeries are thyroid cancer, toxic multinodular goiter, toxic adenomas, local compressive symptoms, Graves' disease that failed or refractory to medical treatment or for whom medical management may not be advised, such as those attempting to become pregnant, and, rarely, cosmesis [5]. In our study, the main indication was a compressional symptom in 35%. A similar indication was reported by Al-Hureibi *et al*. [6]. On the contrary, in Alyahya *et al*. study, neck mass was declared the most common indication [2]. In our study, the mean age was 41.60± 8.21, with the most affected (30.1%) in the age category of 31-40 years. Similarly, in Wondwosen *et al*. study, the mean age for thyroidectomy was 41 ± 12.46, with 59 (29.6%) in the age category of 31-40 years [7].

Factors such as iodine deficiency and bad nutrition may contribute to an increase in the thyroid disease in this age group. Fine needle aspiration biopsy is considered a cost-effective and safe test for evaluating thyroid masses, which may be performed with ultrasound assistance for higher diagnostic accuracy [8]. The current study found that 94.3% of benign thyroid diseases were diagnosed by FNAB, which varies from previous findings in studies such as Al-wageeh *et al*., who found that 66.2% of benign thyroid diseases were diagnosed by FNAB [3]. Fine needle aspiration biopsy showed high accuracy in detecting papillary, medullary, and anaplastic thyroid carcinoma; however, it has lower accuracy and precision in the preoperative diagnosis of FTC. These findings may be explained by follicular carcinoma's encapsulated and highly vascular nature [3]. Thyroidectomy may be performed for several benign and malignant disorders, including thyroid nodules, hyperthyroidism, substernal goiter, thyroid malignancies, and thyroid lymphoma [5]. Total thyroidectomy or hemithyroidectomy with contralateral near-total excision has been suggested in the literature, given the lower recurrence and re-operation rate. In this study, most of our operations, 75.6% were near-total thyroidectomies, similar to Al-Hureibi *et al*. study [6].

Thyroid carcinoma was present in 7.3% of patients, and the most type was FTC in 9 patients. Another study reported 23.3% of thyroid carcinomas [9]. A high rate of thyroid carcinoma was reported by Al-wageeh *et al*., who reported that 28.1% of thyroidectomy specimens showed thyroid carcinoma [3]. This could be interpreted as the total number of cases, the monocentric nature of our study, the different populations, and the geographical distribution of the disease in those studies. Another explanation is that most cancerous patients are preferred undergoing surgery in a referral oncologic center in Sanaa city, where the cost of operation is few. The most prevalent consequences of thyroidectomy were recurrent laryngeal nerve palsy, low calcium levels, and local hematoma [10]. In our study, complications occurred in 19.1% of patients. Intraoperative bleeding was the most typical complication presented in 36 (14.6%) patients, including postoperative hematoma in 10 (4.06%) during admission with respiratory compromise or obstruction that necessitates re-intervention or evacuation. Our explanation for high complications is that the general surgeon performed all the operations. Additionally, most of the mentioned complications are temporary and easy to treat. It was reported that the surgeon's experience was influenced significantly by the incidence of postoperative complications. The higher-volume surgeons had lower complication rates [10]. There were several limitations in the current study. A monocentric and retrospective nature is the main limitation of our study. Some data, such as thyroid gland volume and long postoperative survival rate, were not investigated. A future prospective multicentric survey with a large patient number and longer follow-up is recommended to confirm our result.

## Conclusion

The findings of this study demonstrate that compressional symptoms were the most symptoms of patients who underwent thyroidectomy, the commonest preoperative cytology finding was multinodular goiter, and near-total thyroidectomy was the most performed surgery. The most prevalent complications were intraoperative bleeding. Furthermore, FTC was the most common type of thyroid cancer and was mainly misdiagnosed on FNAB preoperatively.

### What is known about this topic

- *Thyroid disease incidence and prevalence in a community are variables that are affected by a variety of factors;*
- *Although thyroid surgery is the most common and safe operation in the world, it is not without risk of complications and death due to the anatomical structure and physiological function, particularly of the thyroid gland*.

### What this study adds

- *In our population, multinodular colloid goiter is the most common thyroid disease and compression symptoms were the most common reason for thyroidectomy;*
- *Performing fine needle aspiration biopsy initially is more helpful for distinguishing between benign and malignant thyroid nodules;*
- *Intraoperative bleeding was the commonest complication of thyroidectomy in this study*.
